# Pulmonary Targeting of Inhalable Moxifloxacin Microspheres for Effective Management of Tuberculosis

**DOI:** 10.3390/pharmaceutics13010079

**Published:** 2021-01-08

**Authors:** Bhavya Vishwa, Afrasim Moin, D. V. Gowda, Syed M. D. Rizvi, Wael A. H. Hegazy, Amr S. Abu Lila, El-Sayed Khafagy, Ahmed N. Allam

**Affiliations:** 1 Department of Pharmaceutics, JSS College of Pharmacy, Mysuru 570015, India; bhavyavishwa@gmail.com; 2 Department of Pharmaceutics, College of Pharmacy, University of Hail, Hail 81442, Saudi Arabia; afrasimmoin@yahoo.co.in (A.M.); syeddanishpharmacy@gmail.com (S.M.D.R.); a.abulila@uoh.edu.sa (A.S.A.L.); 3 Department of Microbiology and Immunology, Faculty of Pharmacy, Zagazig University, Zagazig 44519, Egypt; waelmhegazy@daad-alumni.de; 4 Department of Pharmaceutics and Industrial Pharmacy, Faculty of Pharmacy, Zagazig University, Zagazig 44519, Egypt; 5 Department of Pharmaceutics, College of Pharmacy, Prince Sattam Bin Abdulaziz University, Al-kharj 11942, Saudi Arabia; e.khafagy@psau.edu.sa; 6 Department of Pharmaceutics and Industrial Pharmacy, Faculty of Pharmacy, Suez Canal University, Ismailia 41552, Egypt; 7 Department of Pharmaceutics, Faculty of Pharmacy, Alexandria University, Alexandria 21521, Egypt

**Keywords:** tuberculosis, microspheres, moxifloxacin, dry powder inhalers, pulmonary drug delivery

## Abstract

In the present study, the objective was to attain a localized lung delivery of an anti-tubercular fluoroquinolone, moxifloxacin (MXF), targeting the alveolar macrophages through a non-invasive pulmonary route using inhalable microspheres as a dry powder inhaler approach. MXF-loaded poly (lactic-co-glycolic acid) (PLGA) microspheres (MXF-PLGA-MSs) were fabricated by solvent evaporation technique and optimized by using a central composite statistical design. The morphology and particle size, as well as the flowability of the optimized microspheres, were characterized. In addition, the aerosolization performance of the optimized formula was inspected using an Andersen cascade impactor. Furthermore, in vivo fate following intrapulmonary administration of the optimized formula was evaluated. The optimized MXF-PLGA-MSs were spherical in shape with a particle size of 3.16 µm, drug loading of 21.98% and entrapment efficiency of 78.0%. The optimized formula showed a mass median aerodynamic diameter (MMAD) of 2.85 ± 1.04 µm with a favorable fine particle fraction of 72.77 ± 1.73%, suggesting that the powders were suitable for inhalation. Most importantly, in vivo studies revealed that optimized MXF-PLGA-MSs preferentially accumulated in lung tissue as manifested by a two-fold increase in the area under the curve AUC_0–24h_, compared to plain drug. In addition, optimized MXF-PLGA-MS sustained drug residence in the lung for up to 24 h following inhalation, compared to plain drug. In conclusion, inhalable microspheres of MXF could be a promising therapeutic approach that might aid in the effective eradiation of tuberculosis along with improving patient adherence to the treatment.

## 1. Introduction

Tuberculosis (TB) is a contagious bacterial disease that is caused by mycobacterium tuberculosis. It ranks among the top 10 worldwide causes of death and is the leading basis of death from a single infectious agent (ranked above HIV/AIDS) [1]. It usually affects the lungs (pulmonary TB) but can similarly affect other locations (extra pulmonary TB). In 2019, the World Health Organization (WHO) reported a global mortality of 1.45 million people (0.25 million non-HIV and 1.2 million with HIV) deaths due to TB [2].

The current first-line six months treatment regimens for drug-susceptible TB are highly effective; however, challenges including the development of severe side effects and/or drug resistance, co-infection with HIV, poor adherence to treatment regimen, or variation in pharmacokinetics may compromise the treatment outcomes [3,4]. Consequently, developing safe and efficient treatment regimens that improve cure rates could reduce mortality/morbidity along with reduction in drug resistance is of utmost importance.

Moxifloxacin, an 8-methoxy quinolone, is highly active against Mycobacterium tuberculosis [5,6], and is currently recommended by WHO for treating multi-drug resistant TB [7]. In addition, it can also be recommended in drug-susceptible TB, in patients who are not capable of tolerating the first-line TB drugs [8]. Recently, many clinical trials have emphasized the therapeutic efficacy of moxifloxacin, either as a part of a multi-drug regimen against multi-drug resistant TB or as a part of novel treatment shortening regimens, with bedaquiline, pretomanid or rifapentine, for treating drug-susceptible TB. Nevertheless, gastrointestinal disturbances, such as nausea and diarrhea, associated with moxifloxacin administration might limit the utilization of moxifloxacin in many clinical settings [9,10]. Accordingly, the search of an alternative route of drug administration other than the oral route is a prerequisite.

Pulmonary delivery of drug has been recently emerged as a non-invasive and attractive approach for the treatment of numerous pathologies, especially those affecting lungs [11,12]. The interest in this approach was sparked by the possible utility of the lung as a gateway for the entry of drugs including proteins and peptides. Pulmonary delivery of drugs exerts potential advantages compared to the oral or parenteral routes. The large surface area of the human lung with its rich blood supply provides ideal conditions for rapid drug absorption into the systemic circulation and ensures a fast onset of drug action [13,14]. In addition, targeted drug delivery to the lungs enhances the therapeutic ratio significantly by decreasing severe systemic side effects and increasing drug therapeutic efficacy [15,16]. Furthermore, the lungs have comparatively low local metabolic activity and, thereby, show no or limited first pass metabolism [17]. Finally, alveolar macrophages can be targeted for management of pulmonary TB [18].

Dry powder inhalers (DPIs) have recently gained a great deal of popularity as an efficient method to achieve drug delivery to the lung [19,20]. DPIs are devices by which a dry powder formulation of the active substance is delivered through the pulmonary route for either local or systemic effect. They offer the advantages of being eco-friendly, compact and portable and exert superior patient adherence and better lung delivery than nebulizers or metered dose inhalers [21]. The particle size of these powder formulations should not be more than 2 μm for the systemic effect and not more than 5 μm for local effect [22]. However, at this size range, inhalable powders show strong interparticulate cohesion, which results in poor powder flowability. Accordingly, to overcome such problems, many approaches have been adopted to improve the aerosolization of powder, such as loading the drug onto inert particulate system such as microparticles or alteration of particle morphology, particle porosity or powder density [23,24].

Particulate-based pulmonary systems have shown considerable potential to improve drug bioavailability and therapeutic efficacy for the deep lung [25,26]. Polymeric microparticles are drug delivery systems with a size range between 1 and 1000 µm. They are mainly composed of biocompatible and biodegradable natural or synthetic polymers such as chitosan, poly (lactic) acid, poly (lactic-coglycolic) acid and poly (lactic-co-lysine graft lysine) [27,28,29]. The utilization of polymeric microparticles as vehicles for pulmonary drug delivery offers potential advantages such as physicochemical stability, higher drug loading efficiency, and, most importantly, specific drug targeting to the site of action [30,31]. Furthermore, the properties of polymeric microparticles such as particle size, morphology, and porosity can be easily manipulated to satisfy the requirement of pulmonary delivery.

In this study, therefore, we aimed to investigate the therapeutic potential of moxifloxacin-loaded microspheres against Mycobacterium tuberculosis infection. Moxifloxacin loaded onto poly (lactic-co-glycolic acid) microspheres were prepared and evaluated for in vitro dry powder inhalation (DPI) performance and in vivo pulmonary pharmacokinetics.

## 2. Materials and Methods

### 2.1. Materials

Moxifloxacin Hydrochloride (MXF) was generously obtained as a gift sample from Micro labs limited (Bengaluru, India). Poly (lactic-co-glycolic acid) (PLGA; LA/GA = 50/50) was kindly obtained from Evonik India Pvt ltd (Mumbai, India). Resazurin sodium salt was procured from Sigma-Aldrich (New Castle, PA, USA). Polyvinyl alcohol (PVA) and dichloromethane (DCM) were used without further purification.

### 2.2. Formulation and Optimization of Microspheres Loaded with MXF

#### 2.2.1. Preparation of MXF-Loaded Microspores

MXF-loaded PLGA microspheres (MXF-PLGA-MSs) were prepared using solvent vaporization method [32]. MXF and PLGA polymer were dissolved in an organic solvent composing of mixture of dichloromethane and methanol (1:1 ratio). Briefly, 20 mg of MXF and 20 mg of PLGA were dissolved in 1mL of organic internal solvent. The organic phase was then slowly dispersed in 4 mL of an aqueous phase containing 2% poly vinyl alcohol with continuous stirring. The resultant emulsion was homogenized for 2 h at a rotational speed of 3500 rpm using high shear homogenizer (T 25 digital ULTRA-TURRAX^®^, IKA^®^-Werke GmbH, Humburg, Germany). Further vaporization of organic solvent was assisted by mechanical stirring at 3500 rpm overnight. The resultant microspheres were collected by centrifugation at 3000 rpm for 30 min and purified by washing with double distilled sterile water for 3 times. The obtained microspheres were freeze dried until subsequent use.

#### 2.2.2. Experimental Design

A 2-factor, 2-level central composite design (Version 12, Stat-Ease Inc., and Minneapolis, MN, USA) was utilized to formulate MXF-loaded PLGA microspheres and to investigate the effect of different formulation variables on product characteristics. A total of thirteen runs were performed. The central composite design enables the study of the effect on formulation characteristics, such as drug loading, entrapment efficiency and particle size, of two independent formulation variables, namely, drug concentration and polymer concentration. The experimental design matrix of the central composite design is summarized in Table 1. It provides a second-order quadratic equation without using complete three-level factorial experiments.

### 2.3. Structural Characterization of MXF-PLGA-MS

#### 2.3.1. Drug Crystallinity Study

The structural characterizations of pure MXF, PLGA polymer, physical mixture of MXF+PLGA and MXF-PLGA-MS was conducted by Advance X-ray powder diffractometer (Bruker AXS GmbH, Karlsruhe, Germany) equipped with a 1D VANTEC position sensitive detector.

#### 2.3.2. Thermal Analysis

Differential scanning calorimetric studies for MXF, PLGA polymer, physical mixture of MXF+PLGA and MXF-PLGA-MS were conducted using SDT 2960 Simultaneous DTA-TGA/DSC-TGA thermal analyzer (TA Instruments, New Castle, PA, USA).

#### 2.3.3. Fourier Transform Infrared Spectroscopy (FTIR)

In order to detect the interaction between drug and polymer, FT-IR spectra of MXF, PLGA polymer, physical mixture of MXF+PLGA and MXF-PLGA-MS were recorded with FTIR spectrophotometer (Perkin Elmer Corporation, Mississippi, MA, USA) between 4000 and 400 cm^−1^. The obtained absorbance spectra were taken and analyzed for additional peak appearance or disappearance of peaks.

### 2.4. Evaluation of Prepared Microspheres

#### 2.4.1. Particle Size

Microsphere particle size and polydispersibility index were determined by dynamic light scattering technique using a Malvern Zen 3600 Zetasizer (Malvern Instruments Ltd., Worcestershire, UK). The measurement was conducted at a 90° scattering angle and 25 °C.

#### 2.4.2. Particle Morphology

The optimized formula was subjected for surface morphology studies by tungsten thermionic emission scanning electron microscope system (Tescan, Brno, Czech Republic). The microsphere samples before filtration step and after air drying were used for scanning electron microscopy (SEM) analysis.

#### 2.4.3. Drug Loading and Drug Entrapment Efficiency

A total of 25 mg of microspheres was grounded and dissolved in 20 mL methanol with the aid of vortex sonicator for 20 min. The drug solution was filtered, and the drug concentration was quantified by high performance liquid chromatography (HPLC) system. The actual loading capacity and the entrapment efficiency expressed in percentage were calculated using the following equations:

$$\%\mathrm{Drug}\;\mathrm{loading}=\left(\frac{\mathrm{weight}\;\mathrm{of}\;\mathrm{drug}\;\mathrm{in}\;\mathrm{microsphere}}{\mathrm{weight}\;\mathrm{of}\;\mathrm{microsphere}}\;\right)\times \;100$$

$$\mathrm{Entrapment}\;\mathrm{Efficiency}\;\left(\mathrm{EE}\%\right)\;=\frac{\;\mathrm{Weight}\;\mathrm{of}\;\mathrm{drug}\;\mathrm{in}\;\mathrm{microsphers}}{\mathrm{Total}\;\mathrm{weight}\;\mathrm{of}\;\mathrm{drug}\;\mathrm{taken}}\times 100$$

##### HPLC Method for MXF Determination

For MXF determination, chromatographic separation was achieved by HPLC (Shimadzu, Kyoto, Japan) equipped with a Kromasil C18 (250 mm × 4.6 mm, 5 μm) column. The mobile phase composed of phosphate buffer and acetonitrile (55:45 *v*/*v*; pH 4.4). The column temperature was kept at 40 °C, and the flow rate was set at 1 mL/min. The wavelength of UV detection was set at 294 nm for MXF. Retention time of MXF was found to be 6.32 min. MXF concentration was estimated from a pre-constructed calibration curve of MXF at various concentrations. The linearity was found in the range of 10–500 μg with correlation coefficient (R^2^) equals 0.999. The precision of the method was verified via intra and inter-day assay, which was less than 2%, while the recovery was 99.67–102.01%. The limit of detection (LOD) and the limit of quantification (LOQ) were found to be 1.98 and 6 μg/mL, respectively.

#### 2.4.4. Determination of Moisture Content

Moisture content in the prepared microspheres was analyzed by Karl Fischer volumetric titration technique (using a Karl Fischer auto titrator) [32]. Briefly, dry methanol was placed in the titration cell containing an electrode, a port for introduction of sample and a port for Karl Fischer reagent and a stirrer. Then, 500 mg of drug-loaded microspheres was dissolved in dry methanol in the titration cell. Measured quantities of Karl Fischer reagent were then added to balance the change in the electrical conductivity, brought about by the introduction of microsphere sample, until an end point was reached, which was estimated potentiometrically using a platinum electrode. The moisture content is calculated by estimating the volume of reagent required to bring back the electrical conductivity to its initial value before the addition of microspheres.

#### 2.4.5. Flow Properties Tests

The Carr’s compressibility index and the Hausner’s ratio were calculated to provide a measure of the flow properties. The Hausner’s ratio and Carr’s index were determined as follows:

Hausner’s ratio = *ρ*_t_/*ρ*_b_

Carr’s index = *ρ*_t_–*ρ*_b_/*ρ*_t_

where *ρ*_t_ and *ρ*_b_ were the tapped density and bulk density of the microspheres, respectively.

Angle of repose method [33] was also measured by falling microspheres through a funnel on the horizontal surface. The diameter and the height of powder cone formed upon falling the microspheres through the funnel were measured. The angle of repose was then determined using the following equation:

Angle of Repose (θ) = tan^−1^ [height of the powder cone (h)/radius of powder cone (r)]

#### 2.4.6. Determination of Aerodynamic Diameter

The aerodynamic diameter of the prepared microsphere was determined using Anderson cascade impactor (Erweka^®^, Nottingham, UK). Cascade impactor determines the aerodynamic properties of aerosol particles by separating the particles on impactor plates with respect to their sizes [34]. Briefly, 50 mg of the prepared microspheres was filled into 7 capsules and inserted in an inhaler device. The inhaler device was then connected to the cascade impactor. The microspheres were then introduced to the cascade impactor at a flow rate of 28.3 L/min for 10 s.

Drug particles deposited on each stage were analyzed by HPLC system. Fine particle dose (FPD), fine particle fraction (FPF), and emitted dose (ED) were calculated as follows [35,36].

Fine particles dose (FPD) = mass of particles on stages 2 through 7;

Fine particle friction (FPF) = fine particle dose/initial particle mass × 100;

Emitted dose (ED) = total particle mass on all stages/initial particle mass × 100.

The geometric standard deviation (GSD) and mass median aerodynamic diameter (MMAD) were determined using an online MMAD calculator.

### 2.5. In Vitro Release Studies

The in vitro release of either free MXF or MXF from PLGA microspheres was conducted in phosphate buffer (pH 7.4) and acetate buffer (pH 4.4) as release media to mimic the intracellular condition of lysosomes and phagosomes in the lung. Briefly, 25 mg of MXF-loaded microspheres was dispersed in 500 mL of a dissolution medium kept at 37 ± 1 °C and paddle speed of 100 rpm. At predetermined time points (1, 2, 4, 8, 12, 24… 360 h), a 1 mL sample was removed from medium and replaced by an equivalent amount of fresh dissolution medium to maintain the sink conditions. The drug concentration was quantified using HPLC system.

### 2.6. Alamar Blue Dye for Testing Anti-TB Activity

Microplate Alamar Blue Assay (MABA) was adopted to assess the susceptibility of Mycobacterium tuberculosis to MXF [37]. Briefly, 100 μL of Middlebrook 7H9 broth containing 3 × 10^5^ CFU/mL of M. tuberculosis (Vaccine strain, H37 RV strain, ATCC No—27294) was added to each well of a 96-well plate along with serial dilutions (ranging from 0.8 to 100 µg/mL) of either free MXF or MXF-PLGA-MS. Outer perimeter wells were filled with 200 μL deionized water to avoid dehydration in test wells. The plate was sealed with parafilm and incubated at 37 °C. Five days post incubation, 25 µL of Alamar Blue reagent and 25 µL of 10% Tween 80 were added to each well and the plate was further incubated at 37 °C. A change in color from blue to pink, in the wells, was observed at 24 h. Blue indicates no growth, whereas pink indicates growth.

### 2.7. In Vivo Studies

#### 2.7.1. Animals

Male Swiss albino mice (5 weeks old, 22–25 g) were supplied by Biogen Laboratory Animal Facility (Bengaluru, India). The animals were housed beneath standardized environments of temperature (25 ± 2 °C) and humidity (50 ± 10%), and they were fed standard laboratory chow and water ad libitum. All animal experiments were performed in agreement with the approval of the Institutional Animal Ethics Committee of JSS College of Pharmacy, Mysuru (Approval number P 1-321/2019).

#### 2.7.2. Plasma Pharmacokinetic Study of MXF-PLGA-MS

A plasma pharmacokinetic study was accrued out to evaluate the systemic bioavailability of MXF-PLGA-MS, compared to free MXF, following pulmonary administration using a “nose-only” inhalation exposure apparatus, which was designed to administer dry powder aerosols to mice (Figure S1) [38]. In this study, mice were divided randomly into two groups of five animals in each group. The first group was treated with free MXF, while the second group was treated with MXF-loaded PLGA microspheres. All treated animals received MXF at a dose of 10 mg/kg. Blood samples (500 μL) were collected into heparinized tubes at different time intervals after drug dosing, and plasma was separated immediately via centrifugation for 15 min at 5000 rpm. Then, 300 μL of plasma samples was mixed with an equal volume of acetonitrile for 30 s and the mixture was centrifuged at 4000 rpm for 5 min. The supernatant was then filtered using a 0.22 µL membrane syringe filter and analyzed for MXF using HPLC system.

#### 2.7.3. Lung Biodistribution Study

For organ biodistribution study, mice were divided randomly into two groups of five animals in each group. The first group was treated with free MXF, while the second group was treated with MXF-loaded PLGA microspheres. To trace drug deposition into lung following the inhalation of either free MXF or MXF-loaded PLGA microspheres, in vivo lung biodistribution study was carried out. At different time points (0.5, 1, 2, 6, 12 and 24 h) post free drug or drug-loaded microsphere inhalation, mice were euthanized, lungs were dissected and washed with phosphate buffer saline (pH 7.4) to remove blood taints or adhered other extra tissues. Lung tissue was then homogenized for 1 min at 10,000 rpm under an ice bath and diluted tissue homogenates were stored in eppendorf tubes at −80 °C until further treatment.

### 2.8. Stability Studies

Accelerated stability studies for the optimized microsphere formulation were conducted for six months under various temperature and relative humidity (RH) conditions. Briefly, microspheres filled in capsules were stored in a glass vials and placed in stability chambers with the following storage conditions: 5 ± 2 °C and ambient RH, 25 ± 2 °C and 60 ± 5% RH, and 40 ± 2 °C and 75 ± 5% RH. Stability indicating parameters such as visual appearance, drug loading and entrapment efficiency (%) were estimated at regular intervals up to 6 months according to the guidelines of International Conference on Harmonization ICH [39,40].

### 2.9. Statistical Analysis

All values are expressed as the mean ± SD. Statistical analysis was performed using unpaired *t*-test (SPSS software version 16; SPSS Inc., Chicago, IL, USA). The level of significance was set at *p* < 0.05.

## 3. Results

### 3.1. Formulation of Moxifloxacin-Loaded PLGA Microspheres (MXF-PLGA-MS)

#### 3.1.1. Central Composite Design Experiment and Response Surface Analysis

MXF-loaded PLGA microspheres were formulated and optimized by the two-factor, two-level central composite design (CCD). The CCD comprises 13 experimental runs. The design matrix of the variables and responses has been described in Table 1. The quantitative effects of the independent variables: drug amount (X_1_) and polymer content (X_2_), on the dependent variables: percentage drug loading (R_1_), percentage entrapment efficiency (R_2_) and particle size (R_3_), were fitted into regression analysis, and second-order polynomial equations were obtained to explain the mathematical relationships between the dependent and independent variables (Equations (1)–(3)).

R_1_ = 5.974+1.278 X_1_ + 0.451 X_2_ − 0.0147 X_1_X_2_ − 0.0083 X_1_^2^ − 0.0116 X_2_^2^ (1)

R_2_ = 15.346 + 6.197 X_1_ + 1.099 X_2_ − 0.278 X_1_X_2_ − 0.0786 X_1_^2^ + 0.0934 X_2_^2^ (2)

R_3_ = 2.05 − 0.0476 X_1_ + 0.136 X_2_ + 0.0004 X_1_X_2_ + 0.0043 X_1_^2^ − 0.007 X_2_^2^ (3)

**Figure 1:**
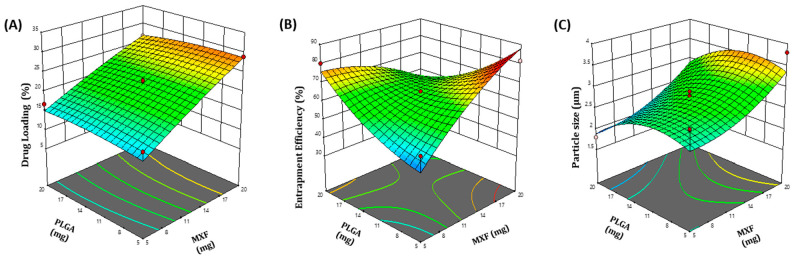
Three-dimensional surface response plots of the estimated effects. (**A**) The effect of drug concentration (X_1_) and polymer content (X_2_) on percent drug loading (R_1_). (**B**) The effect of drug concentration (X_1_) and polymer content (X_2_) on percent entrapment efficiency (R_2_). (**C**) The effect of drug concentration (X_1_) and polymer content (X_2_) on particle size (R_3_).

The magnitude of the studied dependent variables on the investigated responses was explained by three-dimensional (3D) response surface plots (Figure 1). The adequacy and significance of the model were justified by analysis of variance (ANOVA) (Table S1). Analysis of the acquired data showed that the F value of each model has P-value lower than 0.05 (significance level), which indicates that the overall model has significant capacity to explain variation in different response variables; namely, drug loading percentage (Y_1_), entrapment efficiency percentage (Y_2_) and particle size (Y_3_). It is evident that changing drug concentration and polymer content significantly affected the measured formulation parameters (Table 1). Increasing the drug:polymer ratio has proven to increase both percentage drug loading and percentage entrapment efficiency, presumably, by obstructing the leakage of sparingly water soluble MXF to the dispersion media while manufacturing microspheres.

#### 3.1.2. Optimization of MXC-PLGA-MS Formulation

A predictive optimization methodology using the desirability approach was adopted to develop a new formulation with desired responses. The desirable responses were set to fulfil the following criteria: particle size within the aerosolable range (1–5 μm), maximum drug loading and maximum drug entrapment efficiency. The optimized formula for MXF-loaded microspheres was obtained at a drug amount of 19.20 mg and a polymer content of 8.65 mg. The observed drug loading (%), entrapment efficiency (%) and particle size of optimized formula were 22.74%, 78.4% and 3.16 µm, respectively, which were close to the predicted values (22.3%, 78.3% and 3.2 µm) for the optimized formula with a desirability of 0.959.

### 3.2. Characterization of Optimized MXF-Loaded PLGA Microspheres as Dry Powder Inhalation

Previous reports on the development of microspheres as a dry powder inhalation have verified that the physicochemical characteristics of the prepared microspheres, such as particle size, shape of particles, flow property and aerodynamic diameter, are crucial factors that dictate the aerosolization performance of microspheres for inhalation [41,42,43].

#### 3.2.1. Particle Size

Particle size plays an essential role in the phagocytosis of micron-size particles upon pulmonary delivery. Makino and his colleagues demonstrated that polystyrene microspheres with a diameter of 1–6 μm were effectively trapped by rat lung macrophages, compared to smaller size particles [41]. In the same context, Champion et al. reported that rifampicin-loaded PLGA particles with a mean diameter of 1–6 µm showed a comparatively higher uptake by alveolar macrophage cells (NR8383), compared to larger particles [42]. Furthermore, lung macrophages generally do not recognize particles that are either too big or too small. In this study, the mean particle size of the optimized MXF-PLGA-MS formula was 3.16 ± 0.38 μm (Figure S2), which is ideal for targeting MXF to alveolar macrophages for conquering tuberculosis.

#### 3.2.2. Particle Morphology

**Figure 2:**
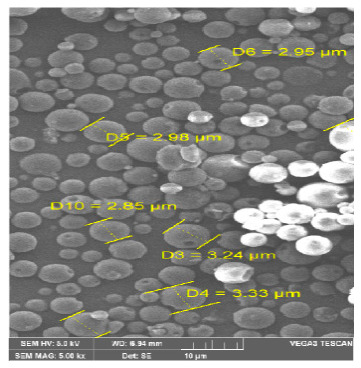
Surface morphology of the optimized formula of a MXF-loaded poly (lactic-co-glycolic acid) (PLGA) microsphere (MXF-PLGA-MS). (Magnification 5000×).

Besides particle size, recent reports demonstrated the impact of particle shape on the uptake of microspheres by macrophages, since particle geometry regulates initial contact and subsequent uptake of particles by macrophages [43,44]. In general, elongated shaped particles are more phagocytosis-resistant than their spherical counterparts [43]. The surface morphology and shape of MXF-PLGA-MS were visualized by scanning electron microscopy (SEM) (Figure 2). It was obvious that the obtained microspheres have a uniform spherical shape with smooth surface, which is expected to facilitate microsphere uptake by alveolar macrophages.

#### 3.2.3. Moisture Content

The dispersion behavior and flowability are two important physicochemical properties of dry-powder inhaler (DPI) pharmaceutical formulations. They are mostly influenced by the moisture content. The presence of free water (moisture) in powder formulations for inhalation can drastically affect the powder flow ability [45] and lead to poor aerosol performance. In this study, moisture content of the optimized MXF-PLGA-MS, determined by Karl Fischer volumetric titration technique, was found to be 4.24 ± 0.43%, which is acceptable for an inhalable microsphere DPI [46].

#### 3.2.4. Flow Properties

Good flow properties are desirable for dry inhalation powder (DPI) handling. Good flowability ensures the accuracy of the dose and, most importantly, allows the fluidization and release of drug powders from the delivery system [45]. In this study, flow properties of the optimized MXF-PLGA-MS were evaluated in terms of Carr’s index, Hausner’s ratio and angle of repose (Table 2). It was perceived that optimized microsphere formulation had better flow properties than free drug. These results indicate that carrier particles, such as microspheres, exert the potential to improve the flow of fine drug particles and might help to obtain uniform filling of fine drug particles into inhalation devices and capsules.

#### 3.2.5. Aerodynamic Diameter

Generally, powders for pulmonary administration should contain aerodynamically dispersed drug particles of 1–5 μm in diameter (most ideally from 1 to 3 μm) to achieve efficient central and deep lung deposition [47]. Table 3 represents the in vitro deposition parameters including MMAD, GSD, FPF, RD and ED. As summarized in Table 3, the mass median aerodynamic diameter (MMAD) of the prepared MXF-PLGA-MS was 2.85 ± 1.04 µm, which was remarkably smaller than that of free MXF (4.85 ± 1.57 µm). In addition, the fine particle fraction (FPF) of drug-loaded microspheres was significantly higher than that of plain MXF. The FPFs of MXF-PLGA-MS and plain drug were 72.78 ± 1.73% and 51.34 ± 146%, respectively. These results indicate that MXF-PLGA-MSs have superior aerosolization properties, compared to free MXF. Of interest, MXF-PLGA-MSs were mainly captured in the lower stages (stages 5 and 6) of the cascade impactor with smaller aerodynamic cut-off diameter (Figure S3), presumably, due to their smaller MMAD and higher FPF. These results confirm the deeper lung accessibility of MXF-PLGA-MS, compared to plain drug.

#### 3.2.6. Drug–Polymer Interaction Studies

##### Crystallinity Study

**Figure 3:**
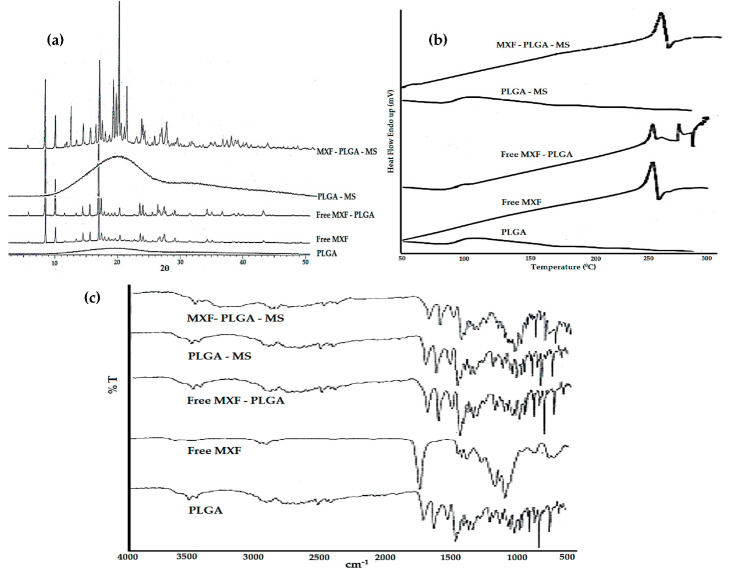
Physicochemical characterization/drug–polymer interaction studies. (**a**) X-ray diffraction spectra, (**b**) differential scanning calorimetry thermograms, (**c**) Fourier transform infrared spectra.

X-ray diffraction (XRD) analysis was conducted to assess any possible changes in the crystallinity due to drug–polymer interaction. The X-ray diffraction pattern for pure MXF, PLGA polymer, physical mixture of MXF+PLGA and MXF-loaded microspheres are shown in Figure 3a. The XRD spectrum of MXF showed sharp crystalline peaks, indicating the crystallinity of moxifloxacin. The lack of crystalline peaks in PLGA, however, indicates the amorphous nature of the polymer. No remarkable changes in either the number or the intensity of peaks were observed in physical mixture of MXF+PLGA and MXF-loaded microspheres, compared to XRD spectrum of pure drug. These results suggest the absence of any incompatibility issues between the drug and the polymer.

##### Thermal Analysis

Differential scanning calorimetry (DSC) was used to identify the possible interaction(s) between MXF and PLGA polymer. DSC thermograms of pure MXF, PLGA polymer, physical mixture of MXF+PLGA are depicted in Figure 3b. The obtained thermogram of MXF showed a sharp endothermic peak at 264.84 °C, corresponding to the melting point of pure MXF. No remarkable shift in the endothermic peak of pure MXF was observed in the physical mixture of MXF+PLGA or MXF-loaded microspheres, indicating the absence of any drug–polymer interactions. However, the presence of PLGA polymer in the physical mixture reduced MXF endothermic peak intensity when compared to pure MXF.

##### Fourier Transform Infrared Spectroscopy (FTIR)

In order to obtain further insight into possible interactions between MXF and PLGA polymer, FTIR spectral analysis was performed and the resulting spectra for pure MXF, PLGA polymer, physical mixture of MXF+PLGA and MXF-loaded microspheres were analyzed for any changes in spectra peaks (Figure 3c). The FTIR spectra of MXF showed characteristic peaks at 1706 cm^−1^ for C=O (stretch), 1320 cm^−1^ for C−N (stretch), 1622 cm^−1^, 1518 cm^−1^ and 1451 cm^−1^ for C=C (stretch aromatic), 1875 cm^−1^ for C−H (bend). FTIR spectra of PLGA polymer showed characteristic peaks at 1749 cm^−1^. The FTIR spectra of the physical mixture (MXF+PLGA) and MXF-loaded microspheres showed no addition or deletion of any peaks from those of the original spectra of the pure drug or the polymer. These results confirm the absence of any interactions between MXF and PLGA.

### 3.3. In Vitro Release Study

**Figure 4:**
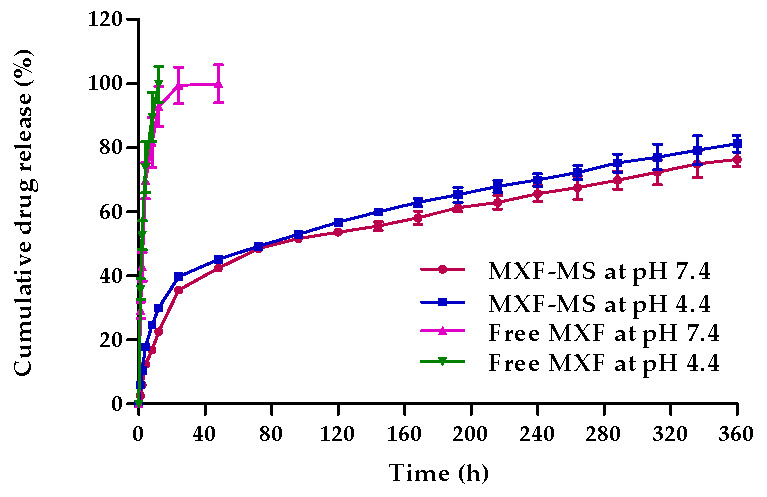
In vitro drug release profile of free MXF and MXF-PLGA-MS in acetate buffer (pH 4.4) and phosphate buffer (pH 7.4).

The in vitro release of free MXF and drugs loaded into PLGA microspheres was conducted in phosphate buffer (pH 7.4) and acetate buffer (pH 4.4) to mimic the intracellular condition of lysosomes and phagosomes in the lung. As shown in Figure 4, free MXF readily released in both phosphate buffer (pH 7.4) and acetate buffer (pH 4.4). The amount of drugs released from the free MXF in acetate buffer saline (pH 4.4) was higher and faster than that in phosphate buffer (pH 7.4), presumably, due to the higher solubility of moxifloxacin in acidic pH than in basic pH. Of interest, drugs loaded into PLGA-MS showed a biphasic release pattern: an initial drug release (up to 20%) at the first 12 h followed by a sustained drug release for up to 360 h. Such biphasic release pattern implies a combination of simple diffusion of MXF from PLGA microspheres and erosion/degradation of the polymer matrix. Initially, the rapid drug release is possibly due to the detachment of the drug adsorbed to the surface of the microspheres and/or the diffusion of drug from the porous voids of the polymer structure. On the other hand, the subsequent slower release of MXF from the microspheres may be attributed to the slower diffusion of the entrapped drug from the microspheres along with the slow degradation/erosion of polymeric microspheres. Of note, no remarkable difference in the in vitro release pattern of MXF from PLGA microspheres was observed at both tested pH values.

### 3.4. Anti-Tubercular Activity of Moxifloxacin

**Figure 5:**
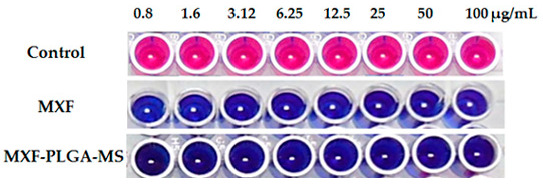
Anti-tubercular activity of free moxifloxacin and MXF-PLGA-MS.

Microplate Alamar Blue Assay (MABA) was adopted to assess the susceptibility of M. tuberculosis to moxifloxacin via monitoring the reducing environment within the living cell. Alamar blue dye is a non-toxic water-soluble redox dye that shows good cell membrane permeability and high stability in culture media [37]. The oxidized form of the dye (resazurin) is blue and non-fluorescent. However, inside the viable cell, resazurin is reduced into pink colored highly fluorescent substance (resorufin). Therefore, growth can be determined by a visual color change from blue to pink. As shown in Figure 5, both free MXF and MXF-PLGA-MS, at all the tested concentrations, exerted a potent antibacterial activity against M. tuberculosis as manifested by inhibiting Alamar blue color change from blue to pink. The minimum inhibitory concentration (MIC) value of MXF, either free or entrapped within PLGA-MS, against M. tuberculosis was 0.8 μg/mL, which was significantly lower than that of either streptomycin, pyrazinamide or ciprofloxacin (Figure S4), indicating the superior anti-tubercular efficacy of MXF compared to first- and second-line anti-tuberculosis drugs.

### 3.5. Stability Studies

Stability studies for MXF-PLGA-MS were conducted according to ICH guidelines. The obtained data suggest that the optimized formula did not show any visual discoloration or altered powder characteristics. In addition, there were non-significant changes in either percentage drug loading or entrapment efficiency of the formulation for up to 6 months at different storage conditions (Table S2). Furthermore, there was a negligible reduction in the drug content at accelerated temperature when compared to the long term or refrigeration temperature (Table S2). Collectively, our results indicate that the optimized MXF-PLGA-MS formula is fairly stable at accelerated and long-term storage conditions.

### 3.6. In Vivo Studies

#### 3.6.1. Pulmonary Pharmacokinetics

**Figure 6:**
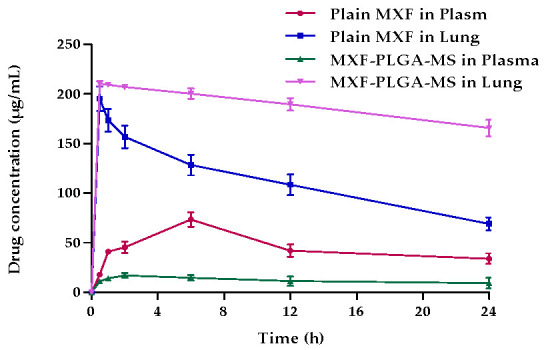
Mean concentration-time profile after inhalation of plain drug and MXF-PLGA-MS in plasma and lung.

In order to verify whether entrapment of MXF within PLGA microspheres could prolong the localized action of MXF in lungs and, thereby, enhance MXF therapeutic efficacy, the in vivo fates of both the plain drug and the optimized formula of MXF-PLGA-MS were evaluated following pulmonary administration. The mean concentration-time profiles following the inhalation of both the plain drug and MXF-PLGA-MS in plasma and lung are represented in Figure 6. As shown in Figure 6, higher drug levels in the lung were observed shortly post inhalation of both plain drug and MXF-PLGA-MS. In addition, PLGA microspheres could maintain higher drug concentrations in lung for prolonged period; up to 24 h post MXF-PLGA-MS inhalation. On the other hand, a significant reduction in plain drug level in the lung was observed shortly post drug administration. Such reduction in plain drug level in the lung was ascribed to the rapid elimination of plain MXF from the pleural cavity into blood circulation as manifested by a mutual increase in drug level in the plasma with a decrease in drug level in the lung.

The mean pharmacokinetic parameters of plain MXF and MXF-PLGA-MS are summarized in Table 4. It is clear that entrapping MXF within PLGA microspheres significantly prolonged drug residence time within lung tissue. The mean residence time (MRT) of MXF-PLGA-MS was 277.39 ± 12.21 h and that of plain drug was 22.42 ± 0.76 h. Most importantly, there was a two-fold increase in AUC_0–24h_ in lung for MXF-PLGA-MS compared with the AUC_0–24h_ of plain drug; the AUC_0–24h_ values were 2686.6 ± 231.4 and 4767.6 ± 230.8 μg·h/mL for plain drug and MXF-PLGA-MS, respectively. The increase in AUC_0–24h_ of optimized microsphere formula could be attributed, at least in part, to the prolonged retention and reduced alveolar clearance of drug-loaded microspheres from lung tissue, compared to plain drug. Similar findings were reported by Mahajan and Gundare [48], who investigated the in vivo fate of the anti-asthmatic agent montelukast sodium loaded onto xyloglucan microspheres following intra-tracheal administration into male Wistar rats. They reported that formulating montelukast sodium in microspheres efficiently prolonged drug residence within lung for up to 6 h post microspheres administration, and, thereby, could prolong drug local action and reduce dosing frequency of montelukast sodium in asthmatic patients.

#### 3.6.2. Bio-Distribution Study

Efficient delivery and sufficient drug retention within the site of action are critical determinants of the effectiveness of targeted drug therapy. Accordingly, a bio-distribution study was carried out to investigate MXF exposure in the organs following intrapulmonary administration of either plain MXF or MXF-PLGA-MS in Swiss albino mice. The percent doses of plain MXF and MXF-PLGA-MS in plasma, lung, kidney, liver and spleen are depicted in Table 5. It was evident that both plain MXF and MXF-PLGA-MS accumulated extensively in lung tissues shortly after drug administration, with 78.1 ± 2.42% and 84.21 ± 2.59% of the administered dose accumulated in the lung 30 min post plain MXF or MXF-PLGA-MS administration, respectively. Nevertheless, in the case of plain MXF, lung accumulation levels were gradually decreased with time, with only 27.63% of administered dose still retained in the lung at 24 h post administration. This might be attributed to rapid elimination of plain MXF from the pleural cavity into blood circulation followed by its rapid re-distribution to reticuloendothelial system organs, mainly liver and spleen, as confirmed by a synchronized increase in drug level in both liver and spleen with the mutual decrease in drug level in the lung. On the other hand, MXF-PLGA-MS restored elevated levels of MXF in lung tissues for prolonged period of time, with up to 78.26 ± 2.19% of the administered dose was detected in the lung tissue at 24 h post microsphere administration. These results imply that drug entrapment within PLGA microspheres efficiently sustains drug action, presumably via reducing alveolar clearance of PLGA microspheres from the lungs and provides a sufficient time gap for the drug to enter into the cell to fight against M. tuberculosis.

Recently, pulmonary delivery of drugs has arisen as a non-invasive and attractive approach for the treatment of several pathologies, especially those affecting lungs [49,50]. Nevertheless, various obstacles including pulmonary clearance mechanisms, metabolic degradation, rapid systemic absorption, and control over drug deposition site have been identified to hinder the development of successful pulmonary drug delivery [50]. Particulate-based pulmonary drug delivery systems provide great opportunities to overcome the aforementioned obstacles. In this study, we adopted microspheres as a particulate carrier for the targeted delivery of anti-tubercular fluoroquinolone drug, MXF, to the lung. The optimized MXF-loaded microspheres (MXF-PLGA-MS) showed a mass median aerodynamic diameter (MMAD) of 2.85 ± 1.04 µm with a fine particle fraction (FPF) of 72.77 ± 1.73%, which reflects a superior aerosolization properties (Table 3) and favors the efficient delivery of inhaled microspheres deeply in the alveolar space. In addition, entrapment of MXF within PLGA microspheres sustained drug release for up to 15 days (Figure 4), and significantly prolonged drug residence time in the lung (Table 4). Collectively, PLGA microsphere might represent a reasonable carrier for eradicating pulmonary tuberculosis while minimizing and/or shortening dosing frequency.

## 4. Conclusions

Moxifloxacin-loaded PLGA microspheres (MXF-PLGA-MSs) were successfully prepared by solvent vaporization technique. The prepared microspheres showed desired properties appropriate for lung delivery as DPI. In addition, in vitro aerosol performance studies of the optimized formulation suggested deep lung deposition of drug. Furthermore, in vivo studies verified the prolonged lung residence time along with the sustained drug release from the fabricated microspheres at the site of infection (lung), raising the possibility of efficiently alleviating tuberculosis along with reducing dosing frequency. To sum up, fabrication of inhalable microspheres of the anti-tubercular fluoroquinolone drug, MXF, can improve the pulmonary targeting and, thereby, might represent a promising candidate for the effective eradication of tuberculosis while improving patient treatment adherence.

## Figures and Tables

**Table 1:** Experimental design matrix of the central composite design with experimental results.

Formula	Coded Values of Independent Values		Actual Values of Independent Values		Response Variables		
	X_1_	X_2_	Drug Amount (mg)	Polymer Content (mg)	R_1_ (%)	R_2_ (%)	R_3_ (μm)
F1	−1	1	5	20	16.80 ± 0.92	80.1 ± 1.57	1.8 ± 1.48
F2	0	0	12.5	12.5	21.67 ± 2.08	65.4 ± 1.22	2.8 ± 1.82
F3	0	0	12.5	12.5	22.89 ± 1.46	65.4 ± 1.87	2.7 ± 1.93
F4	−1.414	0	1.8934	12.5	9.50 ± 1.97	39.5 ± 1.49	2.4 ± 0.78
F5	0	1.414	12.5	23.1066	19.98 ± 1.85	79.2 ± 1.59	1.9 ± 1.38
F6	1	−1	20	5	28.90 ± 1.76	81.5 ± 2.01	3.8 ± 1.46
F7	0	0	12.5	12.5	21.98 ± 1.45	65.4 ± 0.86	2.9 ± 1.67
F8	1	1	20	20	26.50 ± 0.99	45.8 ± 1.06	2.8 ± 1.85
F9	0	−1.414	12.5	1.8934	19.87 ± 1.73	74.8 ± 1.38	1.8 ± 1.67
F10	0	0	12.5	12.5	21.50 ± 2.05	65.4 ± 1.58	2.9 ± 1.86
F11	−1	−1	5	5	15.90 ± 1.09	53.2 ± 0.79	2.9 ± 0.88
F12	0	0	12.5	12.5	22.90 ± 1.81	65.4 ± 1.76	2.7 ± 0.79
F13	1.414	0	23.1066	12.5	31.09 ± 0.94	75.8 ± 2.01	3.8 ± 1.93

X_1_: coded value of moxifloxacin concentration, X_2_: coded value of PLGA concentration. R_1_: percentage drug loading, R_2_: percentage entrapment efficiency, R_3_: particle size.

**Table 2:** Micromeritics properties of the free MXF and optimized MXF-PLGA-MS.

Parameters	Pure MXF	MXF-PLGA-MS
Bulk Density	0.23 ± 0.03 g/mL	1.42 ± 0.11 g/mL
Tapped Density	0.34 ± 0.03 g/mL	1.72 ± 0.16 g/mL
Carr’s Index	32.35	17.44
Hausner’s ratio	1.47	1.21
Angle of Repose (θ)	42 ± 3°	29 ± 2°

Data represent mean ± SD. (*n* = 3).

**Table 3:** Aerodynamic parameters of free MXF and optimized MXF-PLGA-MS.

Parameters	Pure MXF	MXF-PLGA-MS
Recovered dose (RD)	99.14 ± 1.02 μg	90.79 ± 3.41 μg
Emitted dose (ED)	81.86 ± 2.11 μg	82.81 ± 1.67 μg
Fine particles dose (FPD)	50.9 ± 1.05 μg	66.07 ± 1.48 μg
Fine particle friction (FPF (%))	51.34 ± 146	72.77 ± 1.73
Mass median aerodynamic diameter (MMAD)	4.85 ± 1.57 µm	2.85 ± 1.04 µm
Geometric standard deviation (GSD)	1.37 ± 1.98	3.10 ± 1.23

Data represent mean ± SD. (*n* = 3).

**Table 4:** Pharmacokinetic parameters following pulmonary administration of either plain MXF or MXF-loaded microspheres.

Pharmacokinetic Parameters	Plasma		Lung	
	Plain MXF	MXF-PLGA-MS	Plain MXF	MXF-PLGA-MS
C_max_ (μg/mL)	65.92 ± 4.32	16.28 ± 2.41	181.39 ± 31.56	207.39 ± 23.48
T_max_ (h)	4.88 ± 0.42	2.17 ± 0.18	0.13 ± 0.01	0.18 ± 0.01
AUC_0–24h_ (μg/mL*h)	1142.07 ± 101.23	292.71 ± 22.78	2686.68 ± 231.45	4767.57 ± 230.85
MRT (h)	23.25 ± 0.89	33.77 ± 1.11	22.42 ± 0.76	277.39 ± 12.21

**Table 5:** Organ bio-distribution of plain MXF and MXF-PLGA-MS following pulmonary administration.

Formulation	Organ	% Dose Detected					
		30 min	1 h	2 h	6 h	12 h	24 h
Plain MXF	Plasma	7.1 ± 1.21	16.36 ± 2.32	18.15 ± 1.84	29.37 ± 1.92	16.82 ± 1.29	13.58 ± 1.29
	Lung	78.1 ± 2.42	69.35 ± 1.45	62.69 ± 1.32	51.34 ± 1.24	43.38 ± 1.43	27.63 ± 1.48
	Spleen	1.32 ± 1.29	2.63 ± 1.54	4.65 ± 1.33	4.18 ± 2.23	3.54 ± 1.29	4.21 ± 1.59
	Liver	1.87 ± 1.63	5.35 ± 0.54	9.78 ± 0.87	5.29 ± 2.51	15.24 ± 2.15	7.8 ± 1.53
	Kidney	ND *	0.45 ± 0.72	1.25 ± 1.29	1.99 ± 2.54	1.89 ± 1.48	1.23 ± 1.59
MXF-PLGA-MS	Plasma	4.39 ± 1.58	5.62 ± 1.21	6.83 ± 1.29	5.87 ± 1.79	4.53 ± 1.68	3.69 ± 1.25
	Lung	84.21 ± 2.59	83.68 ± 1.11	82.82 ± 2.24	80.12 ± 1.25	79.39 ± 2.17	78.26 ± 2.19
	Spleen	0.45 ± 1.23	0.74 ± 0.32	0.94 ± 0.36	1.55 ± 0.42	0.65 ± 1.59	1.15 ± 0.46
	Liver	1.62 ± 1.41	2.25 ± 1.81	2.78 ± 0.23	3.02 ± 1.11	2.57 ± 2.01	2.27 ± 1.48
	Kidney	ND *	0.92 ± 1.68	1.93 ± 1.11	2.51 ± 1.63	2.99 ± 0.57	0.44 ± 1.01

All data represent the mean ± SD of three independent experiments. * ND: not detectable.

## Data Availability

The data presented in this study are available on request from the corresponding author.

## Supplementary Materials

The following are available online at https://www.mdpi.com/1999-4923/13/1/79/s1, Figure S1: “Nose-only” inhalation apparatus used for administering inhalable microparticles to mice, Figure S2: Particle size distribution of Optimized MXF-PLGA-MS, Figure S3: Moxifloxacin amount deposited into different stages of Anderson Cascade, Figure S4: Anti-tubercular activity of standard drugs; (P) Pyrazinamide, (C) Ciprofloxacin, (S) Streptomycin, Table S1: Model Parameters for the Studied Response Variables, Table S2: Stability Studies of optimized MXF-PLGA-MS.
