# Supplementary material for: Pulmonary Targeting of Inhalable Moxifloxacin Microspheres for Effective Management of Tuberculosis
Source: Pharmaceutics. 2021 Jan 8;13(1):79. doi: 10.3390/pharmaceutics13010079 (PMC7827815; doi:10.3390/pharmaceutics13010079)
Supplement: Supplementary file 1 [file pharmaceutics-13-00079-s001.pdf]

pharmaceutics

MDPI

Supplementary Materials: Pulmonary Targeting of Inhalable Moxifloxacin Microspheres for Effective Management of Tuberculosis

Table S1. Model Parameters for the Studied Response Variables.

Source	Sum of Squares	Df	Mean Square	F-Value	*p*-Value	
			**Drug loading (%)**(Y_1)			
**Model**	361.17	5	72.23	29.52	0.0001	Significant
X_1	354.22	1	354.22	144.74	< 0.0001	
X_2	0.2259	1	0.2259	0.0923	0.7701	
X_1X_2	2.72	1	2.72	1.11	0.3266	
X_1^2	1.52	1	1.52	0.6219	0.4562	
X_2^2	2.96	1	2.96	1.21	0.3075	
			**Entrapment Efficiency (%)**(Y_2)			
**Model**	1614.02	5	322.80	5.04	0.0282	Significant
X_1	256.92	1	256.92	4.01	0.0853	
X_2	0.8304	1	0.8304	0.0130	0.9126	
X_1X_2	979.69	1	979.69	15.29	0.0058	
X_1^2	135.83	1	135.83	2.12	0.1888	
X_2^2	192.20	1	192.20	3.00	0.1269	
			**Particle size (Y_3)**			
**Model**	4.08	5	0.8157	6.37	0.0154	Significant
X_1	1.88	1	1.88	14.69	0.0064	
X_2	0.5076	1	0.5076	3.96	0.0868	
X_1X_2	0.0025	1	0.0025	0.0195	0.8928	
X_1^2	0.4007	1	0.4007	3.13	0.1203	
X_2^2	1.09	1	1.09	8.47	0.0226	

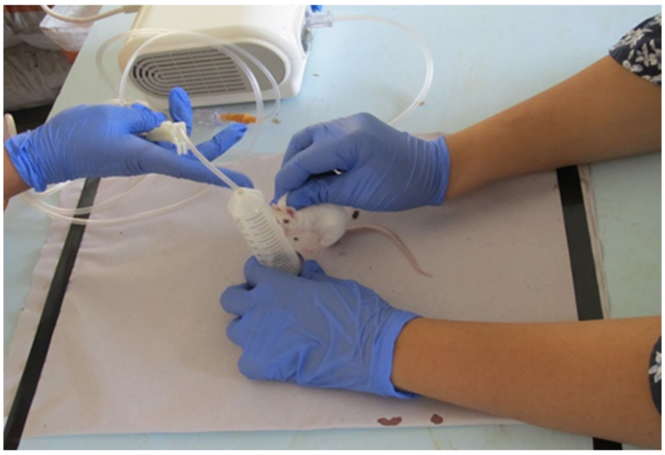

Figure S1. "Nose-only" Inhalation Apparatus Used for Administering Inhalable Microparticles to Mice.

The delivery chamber consisted of a 50-mL plastic centrifuge tube with a hole of ~0.5 cm diameter at a distance of about 2.5 cm from the rim. The powder for inhalation was weighed in the cap. A length of tubing (i.d. ~ 2mm) was inserted into the tube from the apex of the taper (through another orifice) to a clearance of about 2.5–5 mm from the inner

surface of the cap. The tubing was connected to the air pump to admit a turbulent air stream, at a constant rate, for fluidizing the powder for 60 s.

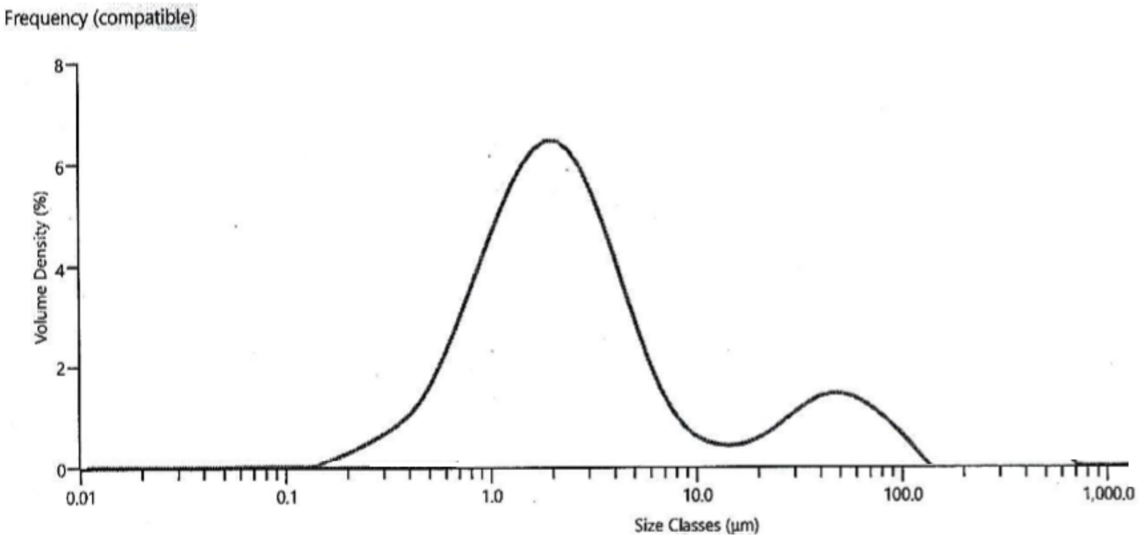

Figure S2. Particle size distribution of Optimized MXF-PLGA-MS.

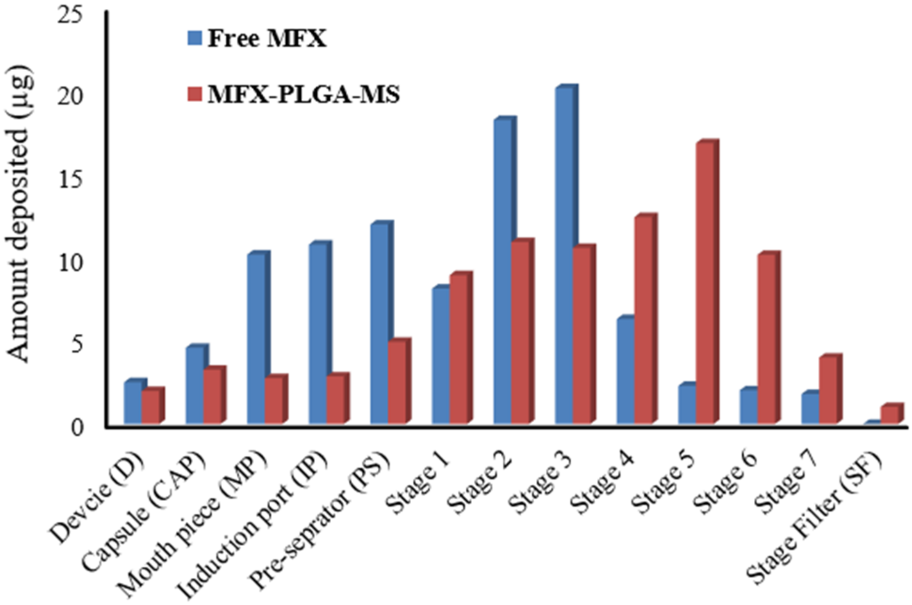

Figure S3. Moxifloxacin amount deposited into different stages of Anderson Cascade.

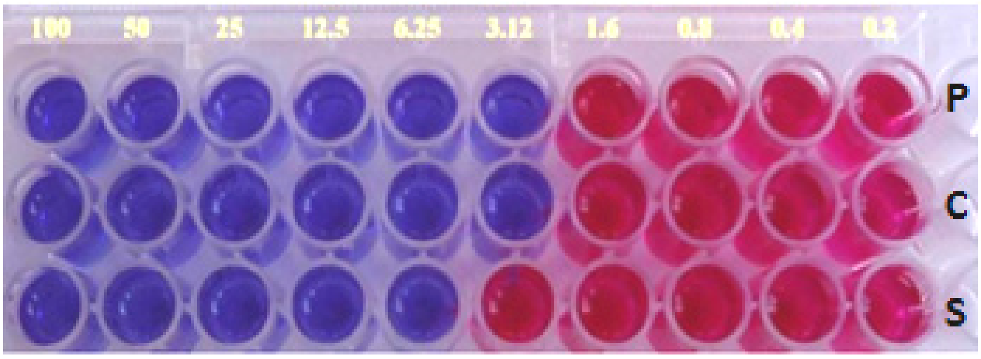

Figure S4. Anti-tubercular activity of standard drugs; (**P**) Pyrazinamide, (**C**) Ciprofloxacin, (**S**) Streptomycin.

Table S2. Stability Studies of Optimized MXF-PLGA-MS.

Stability Condition (Temp./RH)	Time Point	Drug Loading (%)	Entrapment Efficiency (%)
5 ± 2 °C/ambient RH	Initial	21.98 ± 0.23	78.00 ± 1.22
	2 weeks	21.75 ± 0.98	77.68 ± 1.10
	1 month	22.09 ± 1.25	78.12 ± 1.45
	2 months	21.98 ± 2.06	77.89 ± 1.73
	3 months	21.65 ± 1.89	77.93 ± 1.59
	6 months	21.87 ± 1.11	77.55 ± 1.92
25 ± 2 °C/60 ± 5 % RH	Initial	21.98 ± 0.95	78.00 ± 0.99
	2 weeks	21.56 ± 1.65	77.98 ± 1.02
	1 month	21.43 ± 1.89	78.04 ± 1.10
	2 months	21.69 ± 1.76	78.14 ± 1.07
	3 months	21.54 ± 1.32	77.87 ± 1.17
	6 months	21.89 ± 1.25	77.92 ± 1.10
40 ± 2 °C/75 ± 5 % RH	Initial	21.98 ± 1.36	78.0 ± 1.86
	2 weeks	20.98 ± 1.89	78.06 ± 1.11
	1 month	20.16 ± 2.05	77.13 ± 1.33
	2 months	20.07 ± 1.93	77.65 ± 1.75
	3 months	19.87 ± 1.22	76.98 ± 1.32
	6 months	18.16 1.65	73.87 ± 1.65
